# The role of selective attention in value-modulated attentional capture

**DOI:** 10.3758/s13423-026-02964-x

**Published:** 2026-09-18

**Authors:** Francisco Garre-Frutos, Miguel A. Vadillo, Jan Theeuwes, Dirk van Moorselaar, Juan Lupiáñez

**Affiliations:** 1 Department of Basic Psychology, Universidad Autónoma de Madrid, Madrid, Spain; 2 Department of Experimental Psychology and Mind, Brain, and Behavior Research Center (CIMCYC), University of Granada, Granada, Spain; 3 Institute Brain and Behavior Amsterdam, Department of Experimental and Applied Psychology, Vrije Universiteit Amsterdam, Amsterdam, The Netherlands; 4 William James Center for Research, ISPA-Instituto Universitario, Lisbon, Portugal; 5 Faculty of Social and Behavioral Sciences, Experimental Psychology, Helmholtz Institute, Utrecht University, Utrecht, The Netherlands

**Keywords:** Attentional capture, Learning, Reward, Selective attention, Awareness

## Abstract

Stimuli that reliably predict reward can increase their capacity to capture attention. This value-modulated attentional capture (VMAC) is typically viewed as independent of task goals or physical salience, arising from associative learning. However, recent evidence suggests that the awareness of the stimulus-reward contingency may be necessary during the acquisition of such attentional biases, although the underlying mechanism remains unclear. One possibility is that awareness mediates the learning process of VMAC by directing selective attention toward the reward-predictive feature. The present preregistered study tested whether reward-related attentional biases arise primarily from such selective attention, independently of awareness. Participants performed a visual search task in which one of two singleton distractors—one predicting high reward, the other low reward—appeared on a subset of trials. Selective attention to the reward-predictive feature (i.e., distractor color) was manipulated between groups: In some trials, one group reported the reward-predictive distractor’s color, while the other group reported a non-reward-predictive feature (its location). Otherwise, the stimulus–reward contingencies remained identical for both groups. The typical VMAC effect, as measured by slower response times for the high-value compared to the low-value distractor, emerged only in the group that attended to the color. Critically, the previous result cannot be explained by individual differences in awareness. These findings indicate that selective attention to the reward-predictive feature contributes to the acquisition of reward-related attentional biases, independently of awareness about the color-reward contingency.

**Supplementary Information:**

The online version contains supplementary material available at https://doi.org/10.3758/s13423-026-02964-x.

When individuals learn to associate a specific stimulus feature with reward, this stimulus often becomes a stronger distractor in visual search tasks (Anderson et al., 2021; Failing & Theeuwes, 2018). The weight of evidence suggests that the learning process underlying this phenomenon is associative^1^ in nature and thus depends on the history of stimulus–reward pairings (e.g., Bucker & Theeuwes, 2017; Mine & Saiki, 2018). One of the clearest demonstrations of this account is provided by Le Pelley et al. (2015), who used a modified version of the additional singleton task (Theeuwes, 1992, 1994). In their study, participants searched for a target defined by one feature (shape), while a uniquely colored distractor could appear on some trials. Critically, rewards were contingent on the distractor’s color—one color predicted a high reward and the other a low reward magnitude. With these settings, Le Pelley and colleagues found that participants were slower to respond when the high-reward singleton distractor was present compared with when the low-reward singleton distractor appeared, even when slow responses led to the omission of reward.

Once established, this value-modulated attentional capture (VMAC; Anderson et al., 2011a) exhibits strong features of automaticity. For example, it is largely independent of the observer’s goals (Anderson et al., 2011b; Le Pelley et al., 2015), persists under extinction (Garre-Frutos et al., 2024; Watson et al., 2019b), outcome devaluation (De Tommaso et al., 2017; De Tommaso & Turatto, 2021; Watson et al., 2022), and the mere passage of time, with VMAC persisting weeks (Anderson & Kim, 2019)—and even over a year (Anderson & Yantis, 2013)—after the original learning episodes. Furthermore, VMAC is still observed in conditions where attending to the reward-predictive distractor is clearly counterproductive, such as when gazing at the distractor leads to the omission of a potential reward (Failing et al., 2015; Pearson et al., 2016). Indeed, even when participants actively try to prevent capture by the high-value distractor (Pearson & Le Pelley, 2020, 2021), they still attend to it despite being fully aware of the omission schedule (Pearson et al., 2015). Similar conclusions are often drawn about how learning unfolds, as it is frequently assumed that learning can occur implicitly (e.g., Anderson, 2016; Anderson et al., 2021; Failing & Theeuwes, 2018). For instance, whereas early paradigms to study VMAC established feature–reward associations in a separate training stage where the reward-predictive feature is task-relevant (Anderson et al., 2011b), subsequent research has shown that VMAC can be acquired even when the reward-predictive feature is never task-relevant (Mine & Saiki, 2015, 2018), response-independent (Bucker & Theeuwes, 2017, 2018), or even detrimental for performance, as in the paradigm developed by Le Pelley et al. (2015).

Further support for an implicit learning account comes from reports showing that participants often fail to identify the correct feature-reward contingency (Anderson, 2015; Anderson & Yantis, 2013; Grégoire & Anderson, 2019; Theeuwes & Belopolsky, 2012), and sometimes VMAC is observed even among those classified as “unaware” (Bourgeois et al., 2017; Grégoire et al., 2021; Grégoire & Anderson, 2019; Le Pelley et al., 2015). However, this evidence is not as clear-cut as it may seem: other studies have found that only participants aware of the association show VMAC (Failing & Theeuwes, 2017; Garre-Frutos et al., 2025b; Le Pelley et al., 2017; Meyer et al., 2020). An interesting case is the study by Failing and Theeuwes (2017), who conducted six experiments on the boundary conditions of VMAC. They used a procedure similar to that of Le Pelley et al. (2015), but the reward-predictive color distractors were not singletons; instead, they were presented among other colored distractors (e.g., Anderson et al., 2011b). Importantly, Failing and Theeuwes (2017) instructed participants about the color–reward contingencies in all but the last two experiments. In Experiment 5, after four successful replications of the typical VMAC effect, they failed to replicate it when participants were not informed about the contingencies. In Experiment 6, they tried to facilitate learning by reducing the number of distractor colors, and showed that only participants who correctly identified the feature–reward contingencies exhibited a VMAC effect.

While these findings might seem to imply that awareness is necessary for learning, Failing and Theeuwes (2017) argued that reducing the number of distractor colors may have merely facilitated selective attention to the reward-predictive feature, thereby enhancing learning. However, given that VMAC emerged only among participants classified as aware, and selective attention was not manipulated, such a conclusion is not warranted. A subsequent study by Garre-Frutos et al. (2025b) offered a more direct test: when instructions about the contingencies were manipulated between groups, only instructed participants showed evidence of learning. Crucially, because in Garre-Frutos et al. (2025b) the reward-predictive feature was always a salient singleton distractor, selective attention alone cannot explain the results, which suggest that awareness should play a direct role in learning.

Viewed together, this body of evidence may appear contradictory, but it is well established that human associative learning is strongly modulated by instructions and awareness of the contingencies (Lovibond et al., 2011; Lovibond & Shanks, 2002; Mertens et al., 2016; Mertens & Engelhard, 2020; Weidemann et al., 2016). Some theoretical accounts even propose that associative learning could be entirely propositional (Mitchell et al., 2009), making awareness a necessary condition for learning. Still, while VMAC is a clear example of how learning shapes attentional control, the relationship is not one-way: attention also plays a crucial role in learning (Mackintosh, 1975; Niv et al., 2015; Pearce & Hall, 1980). Hence, the interpretation raised by Failing and Theeuwes (2017) is not unreasonable, in that learning may depend on the kind of attention directed toward the predictive feature (Jiménez & Méndez, 1999). Indeed, a similar result is common in other learned attentional biases, such as visual statistical learning, where selective spatial attention to predictive information is widely considered necessary for learning to occur (Duncan et al., 2024; Golan et al., 2024; Jiang & Chun, 2001; Vadillo et al., 2020, 2024).

A parsimonious alternative account is that awareness may influence learning in VMAC indirectly by encouraging selective attention^2^ toward the specific reward-predictive feature of the salient distractor. For instance, although distractors in the additional singleton task capture attention (Theeuwes, 1992, 1994), participants are never explicitly required to selectively attend to any specific singleton feature–either location, shape, or color–as it is not directly task-relevant. In Garre-Frutos et al. (2025b), instructions (and awareness) about stimulus–reward contingencies might direct attention specifically toward the reward-associated feature, potentially making such selective attention both necessary and sufficient for learning. If selective attention is crucial, any manipulation compelling participants to attend to the reward-associated feature (color) rather than reward-irrelevant features (e.g., location) should elicit the learning of VMAC, independently of awareness.

## The present study

Beyond serving as a clear example of how reward history shapes attentional control, understanding the boundary conditions of the VMAC effect is also relevant from broader theoretical and applied perspectives, including debates about how attention and awareness contribute to learning and decision-making. In complex, multidimensional environments, learning and decision-making depend critically on selective attention, insofar as attention constrains what can be learned from the environment (Leong et al., 2017; Niv et al., 2015; Wilson & Niv, 2012). Consistent with this idea, a substantial body of research has linked attentional prioritization to overt choice (Krajbich et al., 2010; Shimojo et al., 2003; Smith & Krajbich, 2019). In this context, recent proposals have suggested that VMAC may bias how individuals sample information from the environment and, ultimately, how they make decisions (Pearson et al., 2022). Such a mechanism may contribute to maladaptive patterns of decision-making across a range of clinically relevant conditions associated with VMAC (Anderson et al., 2021; Le Pelley et al., 2024), underscoring the importance of clarifying the mechanisms and boundary conditions underlying VMAC.

Although VMAC has often been discussed as a form of implicit selection history (Anderson, 2016; Anderson et al., 2021; Failing & Theeuwes, 2018), recent evidence suggests that contingency awareness plays an important role in learning to prioritize signals of value (Garre-Frutos et al., 2025b). However, the functional role of awareness in this process remains unclear. One possibility is that explicit knowledge of the feature–reward contingency directly contributes to learning. Another, not mutually exclusive, possibility is that awareness promotes VMAC by directing selective attention toward the reward-predictive feature, allowing that feature to be actively encoded and bound to reward. If so, selective attention, contingency awareness, and the acquisition of VMAC should be partly dissociable.

In light of the above, this preregistered study tested whether selectively attending to the reward-predictive feature, rather than a reward-irrelevant one, is sufficient to elicit the learning process underlying reward-related attentional biases. We followed the general procedure of Garre-Frutos et al. (2025b), but instead of manipulating instructions, we added a concurrent task designed to direct selective attention to distinct distractor dimensions. In a between-participants manipulation, one group was asked to report the color of the singleton distractor, and the other reported its location. This design contrasted attention to a reward-predictive feature (color) with attention to a reward-irrelevant feature (location), without informing participants of the color–reward association (conceptually similar to Gao & Theeuwes, 2022).

By requiring one group to report the distractor’s color, we forced participants to maintain an active representation of the reward-predictive feature throughout the task, which should facilitate its binding to the reward outcome even in the absence of explicit knowledge of the color–reward contingency. As noted above, instructing participants about the contingency increases both VMAC and awareness of the feature–reward relationship (Garre-Frutos et al., 2025b), possibly because instructions direct attention to the contingency itself (Kattner, 2012; Li et al., 2025; Mertens et al., 2021). By contrast, our manipulation only requires participants to prioritize the reward-predictive feature, without signaling its association with reward. Thus, if prioritizing the reward-predictive feature contributes to the learning of VMAC, then requiring participants to report that feature should enhance VMAC relative to a condition in which they prioritize a reward-irrelevant feature, without necessarily increasing contingency awareness.

Accordingly, we expected the concurrent task to produce a dissociation in VMAC between the two groups. Following our preregistered hypotheses (see https://osf.io/f3bm8), we predicted that the additional-singleton task would elicit the typical attentional-capture effect (H1), that the VMAC effect would be larger in the color-report than in the location-report group (H2$_{1}$), and that it would emerge only among participants attending to color (H2$_{2}$). Finally, we anticipated that this between-group modulation of VMAC would not be explained by individual differences in contingency awareness (H3), which would indicate that selective attention contributes to VMAC independently of such knowledge.

## Methods

### Participants

We performed a simulation-based power analysis (fully reported in the preregistration; https://osf.io/f3bm8) for the critical Group (color vs. location) × Distractor (high- vs. low-value) interaction, based on a similar effect from the linear mixed model (LMM) reported in Garre-Frutos et al. (2025b). Specifically, in Experiment 2, the authors observed a significant interaction between Group (instructions vs. no instructions about the contingencies) and Distractor (high- vs. low-value) on response times (RTs), with only the instructed group showing a significant VMAC effect. Given that the present study extends the same results in similar settings and conditions, we used the same effect size to estimate the required sample size for 80% power to detect the critical interaction in our design. Importantly, given resource and time constraints for data collection, we preregistered two decisions to maximize power: (1) we excluded trials from the first block of the visual search task, as VMAC is typically not observed in this block (Garre-Frutos et al., 2024, 2025a, b); and (2) given that we have our hypothesis about the direction of the effect, we used a one-tailed test (i.e., higher VMAC for the group tasked to report the color vs. location of the distractor) for the critical interaction, as specified in the preregistration, and following our hypotheses (H2$_{1}$). Power was estimated using R (version 4.3.1; R Core Team, 2023) and the *simr* package (Green & MacLeod, 2016). Simulations indicated that 70 participants per group would yield 84% power (α=.05).

To account for potential exclusions, we collected data from 80 participants per group (*N* = 160). Following the preregistered protocol, seven participants were excluded due to low accuracy (< 70%), RTs deviating ±3 SD from the group mean, or poor performance in the concurrent task. Specifically, as described below, participants in the visual search task completed 288 trials, but were required to report the distractor’s location or color on only 12–24 trials (randomly across participants). Rather than setting a fixed accuracy cutoff, we included only those participants performing above chance on the concurrent task to ensure appropriate performance. As preregistered, this threshold was determined using a binomial test assuming a null hypothesis of p=0.5 and a 5% α level. For example, a participant who completed 12 report trials would need at least ten correct responses (p=.036) to meet the threshold, whereas a participant with 18 trials would require at least 14 correct responses (p=.031). Thus, the accuracy cutoff varies between 75% and 83%, depending on the number of report trials completed. After applying this exclusion criteria, the final sample included 153 participants ($n_{\text {color}} = 77$, $n_{\text {location}} = 76$; 117 self-identified as women; $M_{\text {age}} = 21.4$, SD=4.2).

### Stimuli, materials, and procedure

The materials and procedure were based on Garre-Frutos et al. (2025b). The study was conducted online, consistent with prior work using this task (Albertella et al., 2019, 2020; Garre-Frutos et al., 2024, 2025b; González et al., 2025; Le Pelley et al., 2022; Liu et al., 2021; Watson et al., 2020). The task was programmed in jsPsych (de Leeuw et al., 2023) and hosted on JATOS (Lange et al., 2015). To control for differences in participants’ distance from the screen, we scaled stimulus size to screen distance using the virtual chinrest developed by Li et al. (2020). Before starting the main task, participants underwent a calibration procedure to estimate screen distance. They adjusted the size of a rectangle on their screen to match a standard-sized object (e.g., a credit card or driver’s license). Then, they performed a blind spot measurement by covering their right eye and focusing their left eye on a central placeholder while a red circle moved leftward, pressing the space bar when the circle disappeared. This process was repeated five times, and the average was used to estimate screen distance.

**Fig. 1:**
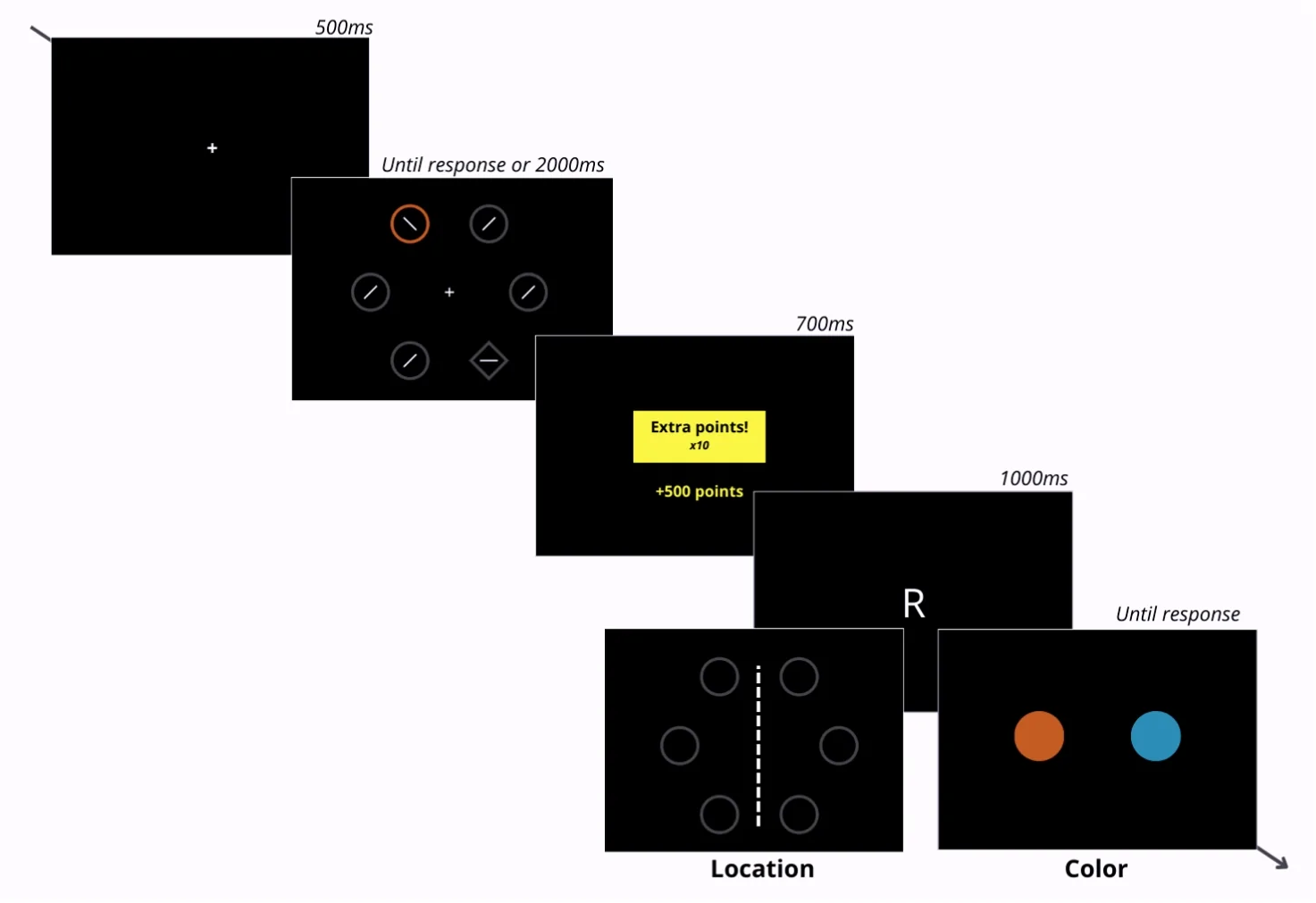
Schematic representation of the task. Example of the sequence of events in the experimental task. Participants could earn points based on performance, and when a high-value singleton appeared in the display, points were multiplied by 10 (a bonus trial). In some trials, participants were presented with the letter ‘R’, which signaled that participants had to report the color or the location (as a function of the assigned group) of the distractor in the preceding trial

As depicted in Fig. 1, each trial started with a central fixation cross, followed by a search display containing six shapes (2.3$^\circ$ - 2.3$^\circ$ visual angle) evenly arranged around an imaginary circle with a diameter of 10.1$^\circ$. The target was a diamond-shaped stimulus containing a line segment oriented either horizontally or vertically, and the non-targets were five circles each containing a line segment tilted 45$^\circ$ to the left or right. On most trials, one of the circles appeared as a uniquely colored singleton distractor (orange/blue or pink/green, counterbalanced), which could be either a high- or low-value color, counterbalanced within each pair. The remaining shapes were gray. The positions of the target and distractor varied randomly in each trial. Participants were instructed to find and report the orientation of the line segment inside the diamond as quickly as possible by pressing the <b> key for horizontal or the <j> key for vertical. The search display remained on screen until the participant responded or 2000 ms had elapsed. Feedback indicating points won or lost was displayed for 700 ms, and the inter-trial interval was 1200 ms.

The task consisted of six blocks of 48 trials (20 high-value singleton trials, 20 low-value singleton trials, and eight distractor-absent trials). Occasionally, participants encountered report trials, indicated by an “R” presented for 1000 ms immediately after the feedback display. Report trials occurred infrequently (two to four per block) with the constraint that at least one appeared in each half of the block; they were restricted to distractor-present trials. In report trials, participants indicated either the side (left or right) of the display on which the distractor had appeared (location-report group) or the distractor’s color (color-report group). In both groups, responses were made with the <c> and <m> keys, which always corresponded to the left and right on-screen response options, respectively. In the color group, either distractor color could appear on the left or right side of the screen during report trials (see Fig. 1). Because the singleton distractor’s position was randomized on every search trial, there was no systematic association between report responses (color or side) and distractor location. In other words, location and singleton color were completely independent in both groups.

Correct responses in the report task earned 2000 points, whereas incorrect responses lost 2000 points; feedback on report performance was provided at the end of each block. In the visual search task, participants received 0.1 points for every millisecond their RTs were below 1000 ms on low-value distractor trials, and the number of points earned was multiplied by ten on high-value distractor trials. No points were earned for RTs exceeding 1000 ms, and incorrect responses resulted in a loss of points equivalent to the potential gain for that trial. With this manipulation, participants distracted by the high-value singleton would acquire significantly fewer points, as the number of points earned in a given trial is directly proportional to performance. After the calibration phase, participants first completed 20 practice trials without any singleton distractors to familiarize themselves with the task. They then performed ten trials with both the visual search task and the report task (after every trial). In this practice stage, the search display was presented for 10 s. After this, participants performed 20 additional trials at normal speed. Subsequently, they entered the visual search task, where instructions emphasized that faster and correct responses result in more points, while incorrect responses result in point losses. Importantly, participants were not informed about the association between the distractor color and reward value. They were only reminded about the distractor in relation to the report task.

At the end of the experiment, participants completed an awareness test to assess their knowledge of the color-reward contingencies using a Visual Analog Scale (VAS; Reips & Funke, 2008)^3^, following the procedure implemented by Garre-Frutos et al. (2025b). They were instructed to respond based on their impressions during the task, avoiding post-hoc interpretations. First, participants rated the extent to which they believed distractor color influenced the likelihood of “bonus trials” (contingency belief), with the scale ranging from 0 (“I don’t believe color makes any difference”) to 100 (“I believe color completely determines the likelihood of bonus trials”). They then estimated the relative proportion of bonus trials associated with each color (contingency awareness). To do this, they were presented with another VAS showing the high- and low-rated distractors with endpoints indicating the percentage of bonus trials estimated to be associated with each color. Participants adjusted a dot along the VAS to represent their estimated likelihood, which updated the displayed percentages accordingly (e.g., a position closer to the high-value color might show a 70% likelihood for the high-value distractor and 30% for the low-value singleton). After providing these ratings, participants indicated their confidence using a confidence VAS ranging from 0 (“no confidence”) to 100 (“very confident”). Finally, participants had the opportunity to provide a free-form qualitative description of any patterns they noticed in the likelihood of bonus trials. This response was collected only for exploratory purposes, but we included a non-preregistered analysis of these verbal reports that replicates the same pattern of results observed in the preregistered analyses (see the “Categorical analysis of contingency awareness” section in the Supplementary Material)

### Data analysis

Unless noted otherwise, all analyses followed our preregistered plan, including data selection criteria, significance thresholds, and the directionality of hypotheses. Data were analyzed using R, version 4.3.1 (R Core Team, 2023) and the R packages *lme4* (Bates et al., 2015b), *betareg* (Cribari-Neto & Zeileis, 2010), *marginaleffects* (Arel-Bundock et al., Forthcoming), and *tidyverse* (Wickham et al., 2019).

#### Report task

We first analyzed performance in the report task. To that end, we compared the two groups on report-task accuracy by fitting a multilevel logistic regression (Jaeger, 2008) with a Group predictor and a by-participant random intercept.

#### Visual search task

The main dependent variable was RTs. We removed RTs considered outliers (RTs < 150 or > 1800 ms; 0.57%), incorrect responses (5.53%), and, following our power analysis, we removed all trials from the first block. Log-transformed RTs were analyzed using an LMM including predictors Singleton (high-value, low-value, absent distractor), Group (color, location), and their interaction. Group was coded using deviation coding. For Singleton, we used repeated contrasts: one contrast for high-value vs. low-value (VMAC effect) and another for low-value vs. absent (attentional capture effect; AC effect). The fixed-effects structure of the LMM had five coefficients: one for Group, two for Singleton (high-value vs. low-value; low-value vs. absent), and two for the interactions between Group and each Singleton contrast. This allowed testing specific hypotheses instead of an omnibus test with no direct theoretical relevance (Schad et al., 2020). Finally, we tested for significance using the preregistered criteria, assuming an α=0.05 and a two-sided contrast. The only exception was the interaction between Group and the VMAC effect, for which we used a one-sided contrast reflecting our directional hypothesis (H2_1_). If a significant Group × VMAC interaction was observed, we computed the conditional VMAC effect for each group. The model’s formula, as implemented in the *lme4* R package (Bates, Mächler, et al., 2015), was as follows:

$$ \log (\text {RT}) \sim \text {Group} \times \text {Singleton} + (\text {Singleton} \ | \ \text {ID}) $$

Regarding the random-effects structure, we first fit the maximum random-effects structure allowed by our design (Barr et al., 2013), including a random slope for the Singleton predictor. In cases of convergence problems or singular fits, we reduced the random-effects structure (Bates et al., 2015a; Matuschek et al., 2017) by dropping the random slope for Singleton. Finally, because between-group comparisons can be attenuated by measurement error (Karvelis & Diaconescu, 2025; Wiernik & Dahlke, 2020; but see Parsons, 2018), we calculated the split-half reliability (Parsons, 2021) of the VMAC effect to inform potential attenuation in the critical between-group comparison.

Finally, although we had no theoretical interest in task accuracy (which is usually near ceiling in similar tasks; Garre-Frutos et al. 2025a, b; Watson et al., 2019b), we analyzed accuracy to rule out a speed–accuracy trade-off. We fitted a multilevel logistic regression, using the same fixed-effects structure as in the LMM for RTs and the same strategy for selecting the random-effects structure.

#### Awareness tests

Participants reported, using two VAS scales (coded 0–100), (1) their belief in the probability of a bonus trial depending on the distractor’s color (*Contingency belief*), and (2) their estimate of the relative likelihood of receiving a bonus trial based on the distractor’s color (*Contingency awareness*). Because both measures are bounded between 0 and 100, we used beta regression (Smithson & Verkuilen, 2006), including a Group predictor to test for mean differences across groups on each VAS. Importantly, as discussed by Smithson and Verkuilen (2006), beta regression assumes known boundaries in the dependent variable but cannot model responses at those exact boundaries. Following their recommendation, we applied a scaling transformation to avoid boundary values: $y^{\prime }=\left[y^{\prime }\cdot \left(N-1\right)+1/2\right]/N$. Each VAS response included an accompanying confidence rating to assess metacognitive validity. To examine the validity of the awareness measures as indicators of explicit knowledge of the feature–reward contingency, we fit the beta-regression models separately for each group, incorporating confidence ratings as predictors. A positive relationship between confidence and performance on the awareness measures would support their validity. Additionally, to evaluate whether the first awareness question predicts the second, we employed the same beta-regression approach.

As preregistered, interpreting H2 as the effect of selective attention on learning required no between-group differences in awareness (H3). To that aim, we tested whether controlling for these individual differences eliminated the Group × VMAC interaction by including the awareness response as a covariate in the RT model described above. To simplify this model, we excluded distractor-absent trials. Because we had two awareness measures, if these measures showed convergent validity (e.g., contingency awareness predicts contingency belief), we used the second contingency awareness as the covariate, which is more similar to the measure employed by Garre-Frutos et al. (2025b).

## Results

### Report task

Accuracy was high ($M_{\text {Accuracy}} = 0.946, \;95\%\,\text {CI}[0.933,$0.959]) and did not differ significantly between groups (*p* = 0.749), suggesting that participants performed well on the secondary task.

### Visual search task

**Fig. 2:**
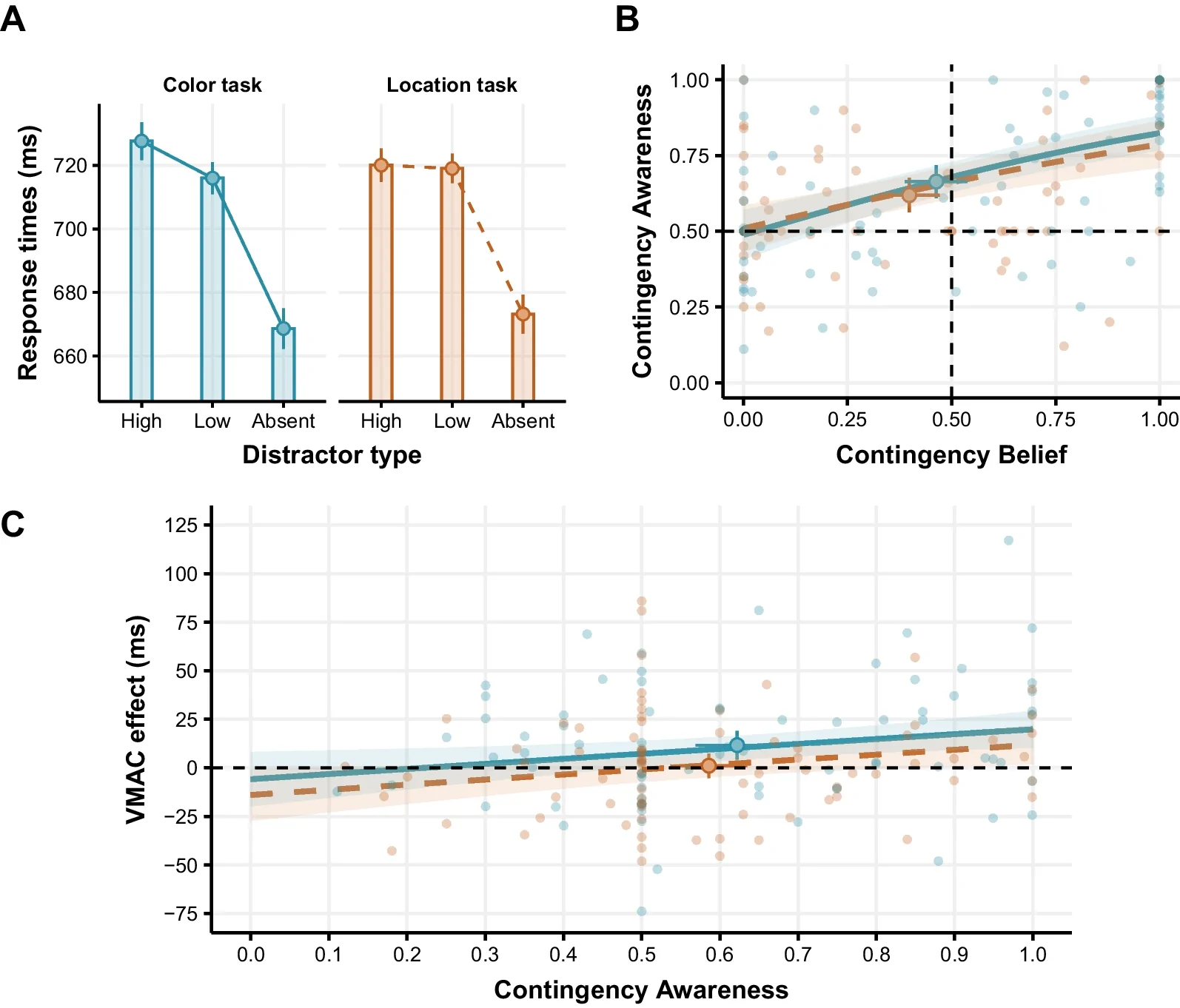
Summary of results. **A**) Mean RTs in the visual search task. *Bars* and *dots* show condition means; *error bars* indicate within-subject 95% CIs (Morey, 2008). **B**) Beta regression predictions for awareness results. *Transparent dots* are individual responses; *lines* and *shaded areas* show model predictions and 95% CIs, and *solid dot points* and *error bars* represent mean predicted responses at the group-level. **C**) RT analysis with contingency awareness as covariate. *Transparent dots* show individual VMAC scores; *lines* and *shaded areas* depict model predictions by task and awareness level. *Solid, large dots* and *error bars* show group means and 95% CIs. Note that both the selective attention manipulation and contingency awareness have independent effects on observed VMAC scores, showing that our results cannot be explained by individual differences in awareness

RTs analysis showed significant VMAC ($$\beta _{\textrm{VMAC}} = 0.008,$$$$\; t_{151} = 2.57, \; p = 0.011$$)^4^ and AC ($$\beta _{\textrm{AC}} = 0.062, \; t_{151} = 17.26, \; p < 0.001$$) effects (Fig. 2A), indicating that participants were slower in the presence of a high- ($$M_{\textrm{High}} = 734.2,\;95\%\,\textrm{CI}[719.7, 748.9]$$) compared to a low-value distractor ($$M_{\textrm{Low}} = 728.4,\;95\%\,\textrm{CI}[713.8, 743.3]$$), and even faster when no distractor was present ($$M_{\textrm{Absent}} = 684.2,\;95\%\,\textrm{CI}[671.1, 697.6]$$). Critically, the Group predictor interacted significantly only with VMAC ($$\beta _{\textrm{VMAC} \times \textrm{Group}} $$$$= 0.006, \; t_{151} = 1.98, \; p = 0.025$$), such that a significant VMAC effect was observed in the color group ($$M_{\textrm{VMAC}} = 10.4,\;95\%\,\textrm{CI}[4.0, 16.8]$$) but not in the location group ($$M_{\textrm{VMAC}} = 1.2,\;95\%\,\textrm{CI}[-5.3, 7.6]$$). No other effects reached significance (*ps* > 0.133).

Given prior evidence that VMAC tends to increase with practice (Garre-Frutos et al., 2024; Le Pelley et al., 2015), we conducted a non-preregistered analysis to examine whether the location-report group showed any evidence of learning later in the task. To this end, we fitted an LMM in the location-report group that included the VMAC effect, Block, and their interaction. The analysis yielded no evidence for either a VMAC effect ($\beta _{\textrm{VMAC}} = 0.002, \; t_{75} = 0.46, \; p = 0.644$) or a VMAC × Block interaction ($\beta _{\textrm{VMAC} \times \textrm{Block}} = 0.004, \; t_{17001} = 1.21, \; p = 0.226$). We also tested whether a VMAC effect was present in the final block of trials, and, again, found no evidence of such an effect ($\beta _{\textrm{VMAC}} = 0.008, \; t_{3396} = 1.05, \; p = 0.293$).

Accuracy in the visual search task was high ($M_{\text {Accuracy}} = 0.953, \;95\%\,\text {CI}[0.948, 0.959]$) and none of the predictors reached significance (*ps* > 0.365), indicating that the above results cannot be explained by a speed–accuracy trade-off.

### Awareness

Neither awareness measure differed significantly between groups (*ps* > 0.224), and the two awareness measures were significantly correlated ($\beta = 0.551,\ z = 6.632,\ p < 0.001$; Figure 2B) with no significant group differences in this correlation (*p* = 0.444). Furthermore, while contingency awareness was positively associated with its corresponding confidence rating ($\beta = 0.579,\ z = 7.197,\ p < 0.001$; no group difference, *p* = 0.409), contingency belief was not (*ps* > 0.231). Overall, these results suggest convergent validity regarding explicit knowledge of the feature–reward contingency, particularly for contingency awareness.

As preregistered, we repeated the analysis of RTs restricted to high- and low-value distractor trials, including contingency awareness and its interaction with VMAC as covariates. This analysis revealed a significant VMAC × Contingency Awareness interaction ($\beta _{\textrm{VMAC} \times \textrm{Awareness}} = 0.004, \; t_{151} = 2.58, \; p = 0.011$), indicating that VMAC was positively associated with contingency awareness (Fig. 2C). Critically, the VMAC × Group interaction remained significant ($\beta _{\textrm{VMAC} \times \textrm{Group}} = 0.003, \; t_{151} = 1.80, \; p = 0.037$) after controlling for individual differences in awareness^5^. Follow-up analyses controlling for awareness revealed that the color group still showed a significant VMAC effect ($M_{\textrm{VMAC}} = 7.2,\;95\%\,\textrm{CI}[0.6, 13.8]$), while the location group did not ($M_{\textrm{VMAC}} = -0.9,\;95\%\,\textrm{CI}[-7.3, 5.5]$). Therefore, even though the VMAC effect was modulated by awareness, the interaction with selective attention cannot be explained away by individual differences in awareness alone. Interestingly, the same pattern emerged when contingency awareness was assessed based on participants’ qualitative reports (see the Supplementary Material).

## Meta-analysis

To contextualize our findings and understand their practical implications, we performed a (non-preregistered) meta-analysis on previous studies employing the same design and settings. Specifically, the present task is an almost exact replication of Garre-Frutos et al. (2024), and the present study derives from Garre-Frutos et al. (2025b). We compared our results with the standardized effect sizes observed in those studies. Additionally, to get a sense of how effect sizes in this specific task design relate to the overall literature, we compared our effect sizes to the meta-analytical estimate from the study of Rusz et al. (2020), a meta-analysis aiming to characterize the size of reward-driven distraction across a wide range of experimental paradigms.

**Fig. 3:**
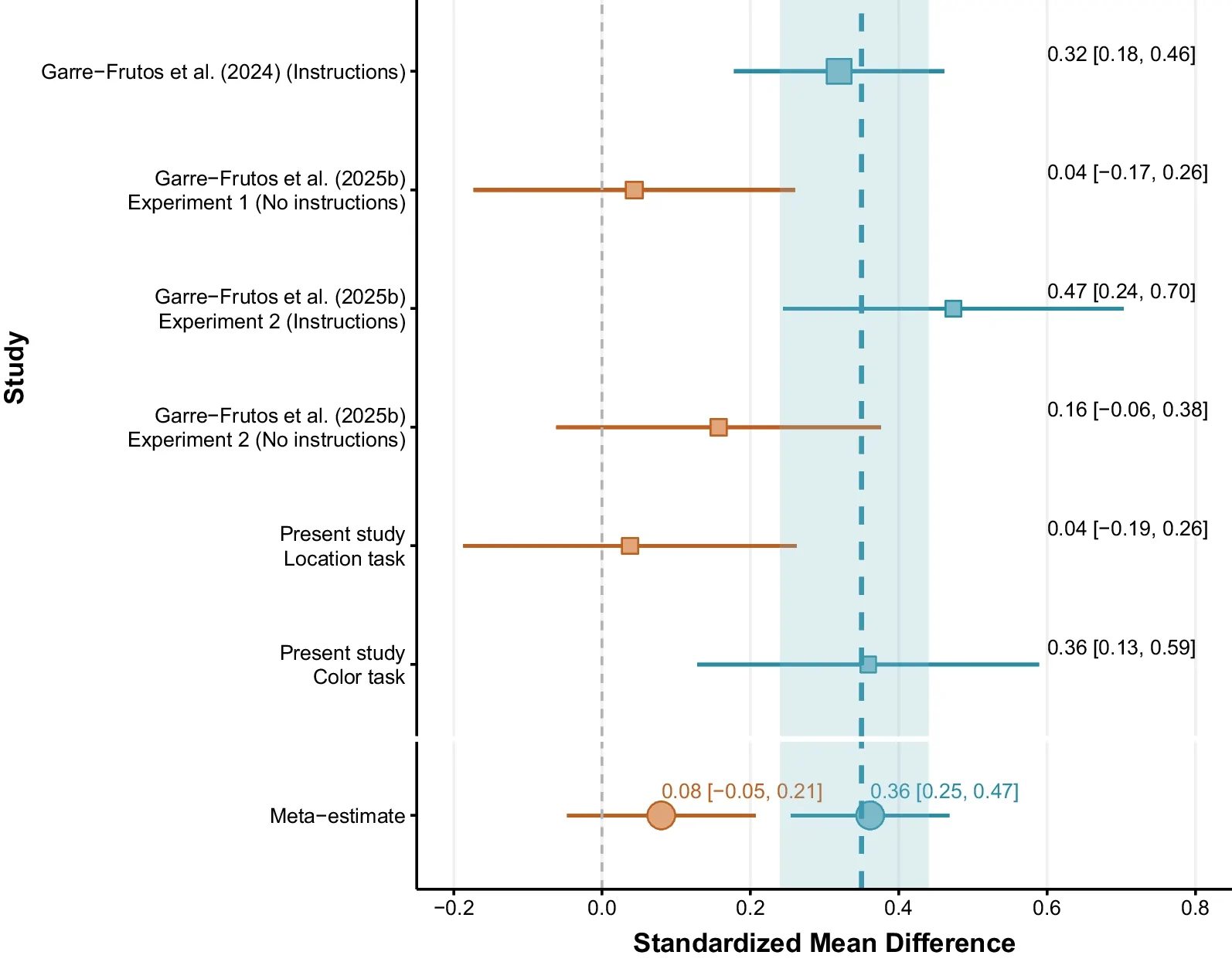
Meta-analysis comparing the present results with prior literature. To illustrate the conceptual similarities between manipulating instructions and selective attention, *blue* indicates that participants received instructions about the feature-reward contingency or selectively attended to color (in the present study). In contrast, *orange* indicates that they did not receive instructions or selectively attended to the location (in the present study). The *blue dashed line* and the *shaded segments* indicate the meta-analytic estimate (standardized mean difference) and the 95% CI of the effect of reward-driven distraction in the broader literature (0.35; Rusz et al., 2020). *Squares* indicate standardized effect sizes for individual studies, with their *size* indicating precision and *error bars* indicating CI. Results from the meta-analysis showed that the effect size observed in the present study for the color group is perfectly consistent with effect sizes observed in the literature and prior studies

Regarding the calculation of effect sizes, as in Rusz et al. (2020), we estimated the standardized mean difference (*SMD*) for the VMAC effect as:

$$ \textrm{SMD} \;=\; \frac{M_{\text {high}} - M_{\text {low}}}{\sqrt{SD_{\text {high}}^{2} + SD_{\text {low}}^{2} - 2r\,SD_{\text {high}}SD_{\text {low}}}} $$

where M and SD are the condition means and standard deviations, and r is the within-participant correlation. The sampling variance was estimated as:

$$ V_{\textrm{SMD}} \;=\; \frac{1}{n} + \frac{\textrm{SMD}^{2}}{2n}. $$

We also estimated the same meta-analytic model using raw differences in RTs to facilitate interpretation of effect sizes within this specific task design (see Supplementary Material). We estimated the sampling variance for raw RTs as:

$$ V_{\textrm{Raw}} \;=\; \frac{SD_{\text {high}}^{2} + SD_{\text {low}}^{2} - 2r\,SD_{\text {high}}SD_{\text {low}}}{n}. $$

We fitted a random-effects model using the *metafor* package (Viechtbauer, 2010), including a moderator coding whether participants were encouraged to attend to the reward-predictive feature—via (or lack of) instructions in prior studies (Garre-Frutos et al., 2024, 2025b) or via task demands (color vs. location reporting) in the present study. This allows us to meta-analytically assess whether manipulating selective attention using a concurrent task had a similar effect as manipulating instructions about the feature-reward contingency.

Results from the meta-analysis (Fig. 3) showed significant differences between the two sets of studies ($\beta = 0.282,\ z = 3.32,\ p < 0.001$) and no significant residual heterogeneity within them (Q(4)=1.974,p=0.740). Interestingly, the VMAC effect was significant only when participants were encouraged to attend to the reward-predictive feature—either via instructions or task demands ($\textit{SMD}=0.36, \ 95\% \ \text {CI} [0.25, \ 0.47]$; $\Delta = 12.37, 95\% \ \text {CI} [8.89, \ 15.84]$)—and not when participants were uninstructed or attended to a reward-irrelevant feature ($\textit{SMD}=0.08, \ 95\% \ \text {CI} [-0.05, \ 0.21]$; $\Delta = 2.19, 95\% \ \text {CI}$[-1.30,5.67]). Notably, the effect size observed for the color group ($\textit{SMD}=0.36, \ 95\% \ \text {CI}[0.13, \ 0.59]$) is fully consistent with the broader literature’s meta-analytic estimate ($\textit{SMD}=0.35, \ 95\% \ \text {CI} [0.26, \ 0.48]$; Rusz et al., 2020), suggesting that only when participants selectively attend to the reward-predictive feature (or are instructed about the contingency) do they exhibit the typical effect size observed in the literature.

## Discussion

In this preregistered study, we investigated the role of selective attention in the learning process underlying VMAC. We manipulated between groups whether participants reported the color or the location of a distractor in a concurrent task, thereby forcing selective attention to a specific feature of the distractor—either reward-predictive or reward-irrelevant. Our results indicate that only participants tasked with reporting the reward-predictive feature showed a significant VMAC effect. Critically, the groups did not differ on any awareness measure, and the modulation of VMAC by task demands remained significant even after controlling for individual differences in awareness. Nevertheless, individual differences in awareness were positively associated with VMAC, suggesting that contingency awareness and selective attention make an independent contribution to the VMAC effect.

Our findings are consistent with the general idea that selective attention modulates how we learn from our environment. Since the influential work of Rescorla and Wagner (1972), most associative learning models have incorporated this idea by assuming that attentional mechanisms can modulate learning (Dayan et al., 2000; Esber & Haselgrove, 2011; Le Pelley, 2004; Mackintosh, 1975; Pearce & Hall, 1980). Several empirical findings align with the notion that attentional mechanisms based on predictability (Mackintosh, 1975) and uncertainty (Pearce & Hall, 1980) not only modulate the rate of learning but also reflect genuine changes in how we prioritize information (Beesley et al., 2015; Le Pelley et al., 2011; for a review, see Le Pelley et al., 2016). Indeed, it is adaptive for an organism to prioritize information that is more likely to be relevant for predicting important outcomes (Watson et al., 2019c). While VMAC may embody one such mechanism, learning what is relevant in a complex, multidimensional environment is likely to require selective attention to reduce its complexity (Niv et al., 2015; Wilson & Niv, 2012). Not surprisingly, taxing attentional resources can impair learning across disparate areas—including Pavlovian learning (Carrillo & Pérez, 2000; Clark et al., 2001; Clark & Squire, 1998; Dawson, 1970; Li et al., 2025; see also Dawson et al., 1982), perceptual learning (Donovan et al., 2015; Szpiro & Carrasco, 2015), instrumental learning (Mastropasqua & Turatto, 2015), implicit sequence learning (Jiménez & Méndez, 1999), and category learning (Kim & Rehder, 2011; Kruschke et al., 2005)—which often formalize the role of selective attention as a gating mechanism whereby attention constrains the input to learning (Leong et al., 2017; Rehder & Hoffman, 2005; Roelfsema et al., 2010; Roelfsema & van Ooyen, 2005).

Similarly, in the context of visual statistical learning, this pattern is also well established: learning is reliably observed only when spatial attention can be directed to spatial or contextual regularities (Duncan et al., 2024; Golan et al., 2024; Jiang & Chun, 2001; Vadillo et al., 2020, 2024). Our results are consistent with this body of evidence, suggesting that selective prioritization of the reward-predictive feature may be necessary for learning^6^. Previous work has shown that value signals that capture attention are reactivated at the moment of reward delivery (Anderson, 2017), and this process has been proposed to operate as a teaching signal that strengthens the perceptual representation of the associated feature (Anderson, 2016, 2019; Rombouts et al., 2015). Because our manipulation required participants to track the reward-predictive feature and report its identity after reward delivery, it is possible that active maintenance of that feature in working memory facilitated the binding between feature and reward representations (Carter et al., 2003; Gilmartin & Helmstetter, 2010; Knight et al., 2004). This interpretation may also help explain why VMAC appears to depend more strongly on factors related to task relevance or contingency awareness (Garre-Frutos et al., 2025b) than other effects typically grouped under the broader umbrella of selection history (e.g., Anderson et al., 2021; Theeuwes et al., 2022; but see Giménez-Fernández et al., 2023), for which the associated elements are somehow task-relevant.

In this vein, the study by Gao and Theeuwes (2022) offers a useful distinction for understanding more generally how learning and attention may interact. Specifically, Gao and Theeuwes (2022) examined whether explicit knowledge of the location in which a singleton distractor was more likely to appear modulated the learning process underlying the distractor-suppression effect, whereby attention is suppressed at locations where a salient distractor is more likely to appear (Wang & Theeuwes, 2018). Using an approach conceptually similar to ours, Gao and Theeuwes (2022) asked participants to report either the location of the target or the location of the singleton distractor, either on every trial (Experiment 1) or every 40 trials (Experiment 2). This manipulation showed that participants tasked with reporting the distractor location were more likely to become aware of the spatial regularity, but learning was not impaired in participants asked to report the target. Importantly, whereas in the present study we manipulated whether attention was directed to a specific feature in the display, Gao and Theeuwes (2022) prompted participants to report the contingency itself, a type of manipulation that is critical for the acquisition of contingency awareness (Kattner, 2012; Li et al., 2025; Mertens et al., 2021). In the case of VMAC, instructing participants about the contingency increases both contingency awareness and VMAC (Garre-Frutos et al., 2025b; see also Mikhael et al., 2021)—possibly because instructions also promoted selective prioritization of the reward-predictive feature. By contrast, in visual statistical learning, where the regularity to be learned is available within the same perceptual episode, only manipulations that prevent participants from attending to the regularity within a search trial appear to disrupt learning (Duncan et al., 2024).

At the same time, manipulations that tax working-memory resources are often found to have little or no effect on learning in visual statistical learning (Gao & Theeuwes, 2020; Vicente-Conesa et al., 2024; but see Vicente-Conesa & Vadillo, 2024 for a critical review), whereas, to our knowledge, there is still no direct evidence about how the same type of manipulation affects the acquisition of VMAC. If selective prioritization of the reward-predictive feature is indeed necessary for learning, then limiting the capacity to represent that feature during the task might also impair learning, even in entirely aware participants. To our knowledge, only one study has manipulated working-memory resources in a VMAC paradigm (Watson et al., 2019a). However, because the contingencies had already been established before the working-memory manipulation was introduced, that study cannot determine how selective attention contributes to the acquisition of VMAC, or how it interacts with awareness during learning.

Interestingly, our results suggest that awareness is related to VMAC not only through selective attention, but also through independent contributions beyond this mechanism. These findings might be taken to imply that the acquisition of VMAC is, at least in part, implicit or functionally dissociable from other mechanisms related to contingency awareness. More specifically, awareness may influence not only how the association is learned, but also how it is expressed. For example, propositional knowledge might trigger learning through a different mechanism (Dayan & Berridge, 2014; Garre-Frutos et al., 2026; Mitchell et al., 2009; Pauli et al., 2019), or amplify VMAC for reasons unrelated to learning—such as strategically directing attention to the high-value distractor (Doyle et al., 2025; Gottlieb et al., 2014; Mahlberg et al., 2025) or by introducing a subtle speed–accuracy trade-off (Garre-Frutos et al., 2025a). Understanding the specific mechanism linking VMAC to awareness is important for assessing both the validity and the practical implications of this attentional bias in psychopathology and decision-making (Anderson et al., 2021; Le Pelley et al., 2024; Pearson et al., 2022).

In summary, our study shows that the relationship between awareness and VMAC may depend on the selective prioritization of the reward-predictive feature. Such prioritization may be necessary to bind the feature effectively to its reward outcome, thereby strengthening its perceptual trace and increasing its subsequent capacity to capture attention.

## Supplementary Information

Below is the link to the electronic supplementary material.

Supplementary file 1 (pdf 36 KB)

## Data Availability

All materials, data, and scripts are publicly available at https://osf.io/ezcrn and https://github.com/franfrutos/vmac_selective, and the preregistered methods and analyses (https://osf.io/f3bm8) were registered before data collection.

## Footnotes

1 We use the term *associative* in a broad sense. The learning process underlying VMAC is often described as Pavlovian, but the reward-predictive distractor can also acquire instrumental properties—particularly in response-time versions of the task, where attending to it carries a performance cost (Heck et al., 2025). Because our question concerns whether prioritizing the reward-predictive feature contributes to the acquisition of these biases, our argument is largely independent of how this learning is framed.

2 Here, and throughout the paper, we use *selective attention* in a broad mechanistic sense to refer to the selective prioritization of information for guiding behavior, whether operating over perceptual input or over internal representations maintained in working memory (Chun et al., 2011; van Ede & Nobre, 2023; Kiyonaga & Egner, 2013).

3 We used a continuous awareness measure because it has been proposed to be more sensitive to low-confidence or partial knowledge than simple categorical measures (Giménez-Fernández et al., 2023; Lovibond & Shanks, 2002; Vicente-Conesa et al., 2023). However, this decision should not be taken to imply that we define contingency awareness as graded or continuous in nature. As described below, we also report a complementary categorical analysis based on participants’ open-ended reports, which yielded similar conclusions (see the Supplementary Material).

4 Split-half reliability of the VMAC effect was low ($r_{\text {sb}} = 0.27, \;95\%\,\text {CI}[0.09, 0.44]$) compared to previous studies using this task Garre-Frutos et al. (2024, 2025b), maybe because of the dual-task design.

5 As preregistered, given that both awareness measures showed convergent validity, we used contingency awareness as the covariate (as in Garre-Frutos et al., 2025b). However, as a (non-preregistered) robustness test, we showed that the results remain unchanged if we use contingency belief, or even if we multiply each awareness measure by its corresponding confidence rating (*ps* < 0.031).

6 Although we interpret the present findings to suggest that selective attention influences learning, one could also argue that our manipulation affected only the expression of that learning. In other words, participants might have learned something, but the expression of that learning was impaired. However, prior work in related literature, such as visual statistical learning (Duncan et al., 2024; Golan et al., 2024) and contextual cueing (Jiang & Chun, 2001; Vadillo et al., 2020, 2024), provides little evidence for robust latent learning when attention to the relevant regularities is prevented. Likewise, VMAC has been observed in conditions that are difficult to reconcile with an “impaired expression” account, such as increased working-memory load (Watson et al., 2019a). We therefore consider this interpretation unlikely, although future studies could test it more directly by including a test phase without any concurrent secondary task.

## Declarations

### Open Practice Statement

This experiment complies with the TOP guidelines. All materials, data, and scripts are publicly available at https://osf.io/ezcrn and https://github.com/franfrutos/vmac_selective, and the preregistered methods and analyses (https://osf.io/f3bm8) were registered before data collection. We report how we determined our sample size, data exclusions, manipulations, and measures in the study.
