# Supplementary material for: The role of selective attention in value-modulated attentional capture
Source: Psychon Bull Rev. 2026 Sep 18;33(7):242. doi: 10.3758/s13423-026-02964-x (PMC13588852; doi:10.3758/s13423-026-02964-x)
Supplement: Supplementary file 1 [file 13423_2026_2964_MOESM1_ESM.pdf]

Supplementary Material to accompany

The role of selective attention in value-modulated attentional capture

Francisco Garre-Frutos[1], Miguel A. Vadillo[1], Jan Theeuwes[2,3], Dirk Van Moorselaar[4], and Juan Lupiáñez[5]

[1]Department of Basic Psychology, Autonomous University of Madrid, Madrid, Spain

[2]Institute Brain and Behavior Amsterdam, Department of Experimental and Applied Psychology, Vrije Universiteit Amsterdam, Amsterdam, the Netherlands

[3]William James Center for Research, ISPA-Instituto Universitario, Lisbon, Portugal

[4]Faculty of Social and Behavioral Sciences, Experimental Psychology, Helmholtz Institute, Utrecht University, Utrecht, the Netherlands

[5]Department of Experimental Psychology and Mind, Brain, and Behavior Research Center (CIMCYC), University of Granada, Granada, Spain

Author Note

Correspondence concerning this article should be addressed to Francisco Garre-Frutos, Department of Basic Psychology, Autonomous University of Madrid, Madrid, Spain. Email: francisco.garre@uam.es

Meta-analysis for the raw VMAC effect

Here, we present the graphical result for the meta-analysis presented in the *Meta-analysis* section of the main text, using the raw differences in reaction times for the high- vs. low-value distractor, and the sampling variance formula described in the same section.

Figure S1

Meta-analysis for the raw VMAC effect

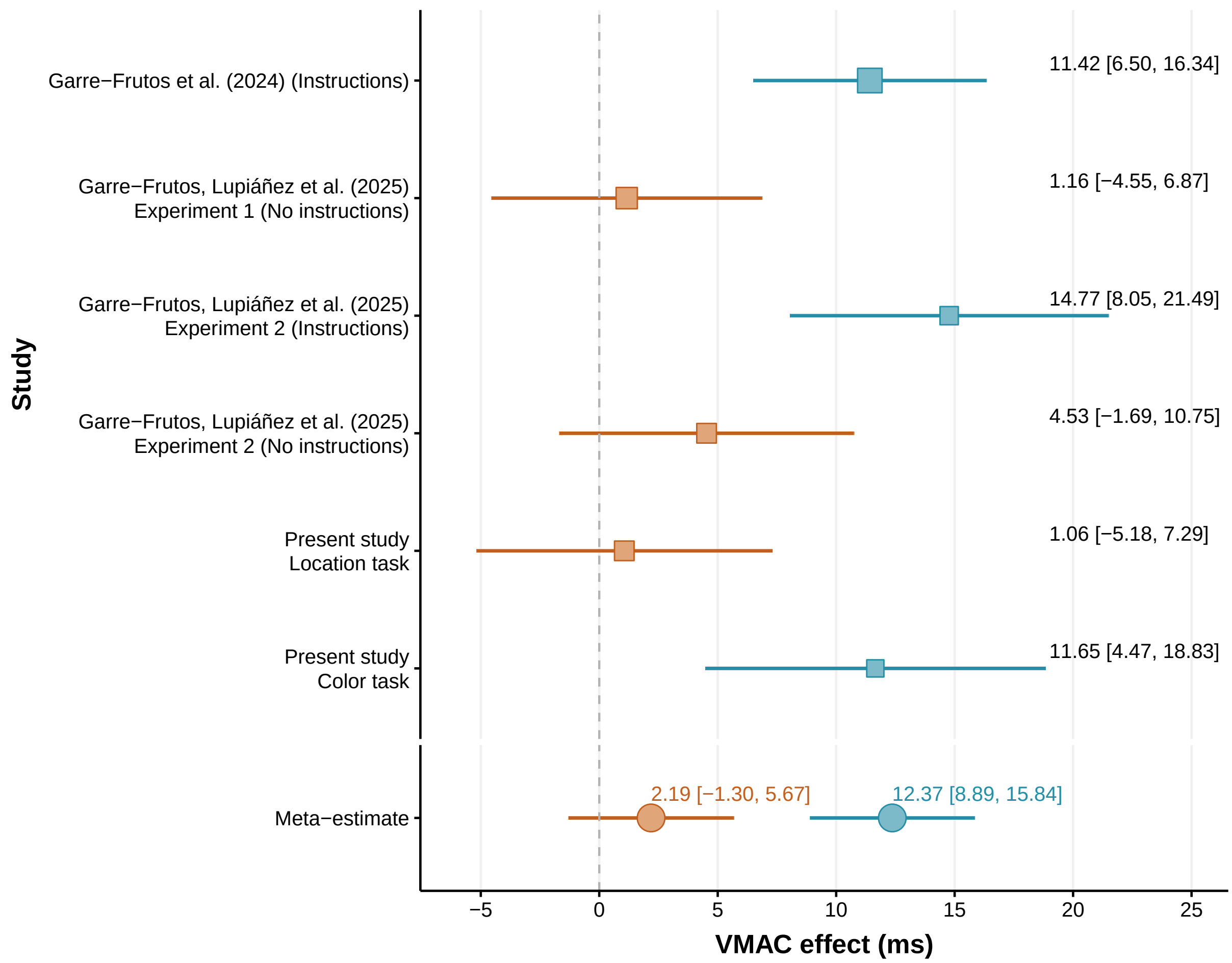

Categorical analysis of contingency awareness

To complement the preregistered continuous awareness analyses reported in the main text, we conducted an additional exploratory analysis based on the open-ended report collected at the end of the experiment. This report asked participants to describe, in their own words, any pattern they noticed in the relation between singleton distractor color and the occurrence of bonus trials.

For the present analysis, participants were classified as *aware* only when they explicitly described the correct color-reward contingency, that is, when they clearly indicated that the high-value distractor color was associated with bonus trials. Participants were classified as *unaware* when they did not describe any color-reward relationship in their report. After applying the preregistered exclusion criteria, this qualitative coding yielded 37 aware participants (20 in the color group and 17 in the location group) and 116 unaware participants (57 in the color group and 59 in the location group).

This qualitative classification was consistent with the continuous contingency-awareness measure reported in the main text. Participants classified as aware gave contingency-awareness estimates that were clearly above chance in both the color group ($M_{\text{Awareness}} = 0.889,\ 95\%\,\text{CI}[0.844, 0.934],\ z = 16.89,\ p < 0.001$) and the location group ($M_{\text{Awareness}} = 0.851,\ 95\%\,\text{CI}[0.790, 0.912],\ z = 11.31,\ p < 0.001$). In contrast, participants classified as unaware performed approximately at chance in both groups (color: $M_{\text{Awareness}} = 0.551,\ 95\%\,\text{CI}[0.494, 0.608],\ z = 1.77,\ p = 0.077$; location: $M_{\text{Awareness}} = 0.542,\ 95\%\,\text{CI}[0.486, 0.598],\ z = 1.47,\ p = 0.142$). Likewise, the probability of being classified as aware did not differ significantly between selective-attention groups ($\beta_{\text{Group}} = -0.197,\ z = -0.52,\ p = 0.603$), with predicted probabilities of $M_{\text{Aware}} = 0.260,\ 95\%\,\text{CI}[0.162, 0.358]$ in the color group and $M_{\text{Aware}} = 0.224,\ 95\%\,\text{CI}[0.130, 0.317]$ in the location group.

We then repeated the RT analysis on high- and low-value distractor trials, including awareness category as an additional between-participants factor. This analysis revealed a significant VMAC by group interaction ($\beta_{\text{VMAC}\times\text{Group}} = 0.014,\ t_{28850} = 2.25,\ p = 0.025$) and a significant VMAC by awareness-category interaction ($\beta_{\text{VMAC}\times\text{AwarenessCategory}} = 0.016,\ t_{28850} = 2.68,\ p = 0.007$), but no three-way interaction ($\beta_{\text{VMAC}\times\text{Group}\times\text{AwarenessCategory}} = 0.007,\ t_{28850} = 0.61,\ p = 0.544$). Follow-up comparisons showed that the color group displayed a significant VMAC effect both among aware participants ($M_{\text{VMAC}} = 20.08\,\text{ms},\ 95\%\,\text{CI}[10.31, 29.85],\ z = 4.03,\ p < 0.001$) and among unaware

participants ($M_{\text{VMAC}} = 6.67\,\text{ms},\ 95\%\,\text{CI}[0.46, 12.87],\ z = 2.11,\ p = 0.035$). In contrast, the location group did not show a significant VMAC effect either among aware participants ($M_{\text{VMAC}} = 8.34\,\text{ms},\ 95\%\,\text{CI}[-2.67, 19.35],\ z = 1.48,\ p = 0.138$) or among unaware participants ($M_{\text{VMAC}} = -0.71\,\text{ms},\ 95\%\,\text{CI}[-6.69, 5.28],\ z = -0.23,\ p = 0.817$). Overall, this exploratory analysis converges with the preregistered analysis.
